# Non-conscious visual cues related to affect and action alter perception of effort and endurance performance

**DOI:** 10.3389/fnhum.2014.00967

**Published:** 2014-12-11

**Authors:** Anthony Blanchfield, James Hardy, Samuele Marcora

**Affiliations:** ^1^Institute for the Psychology of Elite Performance (IPEP), School of Sport, Health and Exercise Sciences, Bangor UniversityBangor, Gwynedd, UK; ^2^Endurance Research Group, School of Sport and Exercise Sciences, University of KentChatham, Kent, UK

**Keywords:** perception of effort, psychobiological model, endurance performance, subliminal, affect, action and inaction

## Abstract

The psychobiological model of endurance performance proposes that endurance performance is determined by a decision-making process based on perception of effort and potential motivation. Recent research has reported that effort-based decision-making during cognitive tasks can be altered by non-conscious visual cues relating to affect and action. The effects of these non-conscious visual cues on effort and performance during physical tasks are however unknown. We report two experiments investigating the effects of subliminal priming with visual cues related to affect and action on perception of effort and endurance performance. In Experiment 1 thirteen individuals were subliminally primed with happy or sad faces as they cycled to exhaustion in a counterbalanced and randomized crossover design. A paired *t*-test (happy vs. sad faces) revealed that individuals cycled significantly longer (178 s, *p* = 0.04) when subliminally primed with happy faces. A 2 × 5 (condition × iso-time) ANOVA also revealed a significant main effect of condition on rating of perceived exertion (RPE) during the time to exhaustion (TTE) test with lower RPE when subjects were subliminally primed with happy faces (*p* = 0.04). In Experiment 2, a single-subject randomization tests design found that subliminal priming with action words facilitated a significantly longer TTE (399 s, *p* = 0.04) in comparison to inaction words. Like Experiment 1, this greater TTE was accompanied by a significantly lower RPE (*p* = 0.03). These experiments are the first to show that subliminal visual cues relating to affect and action can alter perception of effort and endurance performance. Non-conscious visual cues may therefore influence the effort-based decision-making process that is proposed to determine endurance performance. Accordingly, the findings raise notable implications for individuals who may encounter such visual cues during endurance competitions, training, or health related exercise.

## General introduction

The psychobiological model of endurance performance (Marcora, 2008; Marcora and Staiano, 2010), based on motivational intensity theory (Brehm and Self, 1989; Wright, 2008), proposes that the point at which people stop endurance exercise (i.e., exhaustion) is determined by perception of effort and potential motivation. Perception of effort is the conscious sensation of how hard, heavy and strenuous a physical task is (Marcora, 2010) whilst potential motivation is the highest effort a person is willing to exert in order to succeed in a task (Brehm and Self, 1989). Hence, when the effort required by endurance exercise is perceived to exceed potential motivation, or when perception of effort is so extreme that continuing the task seems impossible, the person consciously decides to stop exercising.

According to this effort-based decision-making model, any factor that influences perception of effort and/or potential motivation influences endurance performance, even when the physiological capacity to perform endurance exercise is unchanged. This proposal is in contrast to the muscle fatigue model of endurance performance in which exhaustion is thought to be caused by central and/or peripheral muscle fatigue (Allen et al., 2008; Amann and Dempsey, 2008; MacIntosh and Shahi, 2011) as well as the central governor model which proposes that the subconscious regulation of neural recruitment of locomotor muscles exists to avoid conscious override that may damage the human (Noakes, 2000; St Clair-Gibson and Noakes, 2004). Experimental evidence that conscious psychological manipulations like motivational self-talk (Blanchfield et al., 2014), placebo (Beedie et al., 2006), and competition (Wilmore, 1968) can increase time to exhaustion (TTE) supports the psychobiological model of endurance performance and provides strong evidence against both the muscle fatigue model of endurance performance (Allen et al., 2008; Amann and Dempsey, 2008; MacIntosh and Shahi, 2011) and the existence of a central governor that subconsciously regulates neural recruitment of locomotor muscles based on afferent feedback about the physiological condition of the body (interoception) (Craig, 2002) and the anticipated safe duration of endurance exercise (St Clair-Gibson and Noakes, 2004).

The proposal that the termination of endurance exercise is a conscious decision determined by perception of effort and potential motivation does not exclude the possibility that endurance performance may be influenced non-consciously. In fact, research over the last three decades has illustrated a variety of contexts in which human behavior can be altered by non-conscious psychological manipulations. Of particular relevance to the psychobiological model of endurance performance are studies showing that subliminal reward priming (Pessiglione et al., 2007; Bijleveld et al., 2010), non-consciously activated goal pursuit (Bargh et al., 2001), subliminal affective priming (Silvestrini and Gendolla, 2011b), and non-consciously activated motivation (Banting et al., 2011) can influence effort. Some of these non-conscious psychological manipulations have been shown to influence effort and performance during physical tasks employing small muscle groups (Pessiglione et al., 2007; Aarts et al., 2008; Radel et al., 2009), cycling exercise (Banting et al., 2011) and rowing exercise (Hodgins et al., 2006). However, the effects of priming with subliminal visual cues on perception of effort and performance during whole-body endurance exercise are currently unknown.

The capacity of the non-conscious perceptual system is considerably large in humans when compared to the limited capacity to attend to conscious information (Dijksterhuis and Nordgren, 2006). Hence, only a trivial amount of momentary information is brought to conscious attention. This means that the majority of this momentary information is processed non-consciously (Merikle et al., 2001). Furthermore, this non-consciously processed information can influence human behavior in a manner that resembles conscious awareness of the same information (Bargh et al., 2001; Pessiglione et al., 2007).

Approximately 90% of the human capacity to process non-conscious information is occupied by the visual system (Dijksterhuis and Nordgren, 2006). Consequently, non-conscious visual cues in particular may have a substantial effect on human behavior. In conjunction with this, a variety of visual cues exist within sporting environments. These range from the words and pictures that are displayed on advertisement hoardings to the facial expressions of competitors, team-mates, and even spectators. Indeed, research has demonstrated that tournament favorites are more prone to choking when a trophy is on display during a competitive final (Bijleveld et al., 2011). The possibility that these visual cues may non-consciously impact upon perception of effort and performance during whole-body endurance exercise therefore has considerable implications for endurance athletes.

Interestingly, non-conscious manipulation of visual cues related to affect and action in the form of subliminal priming has been shown to influence effort during cognitive tasks within the framework of motivational intensity theory. For instance, individuals subliminally primed with happy faces exerted greater effort during challenging cognitive tasks in comparison to when primed with sad faces (Silvestrini and Gendolla, 2011b). Furthermore, subliminal priming with action words enhanced participant willingness to exert effort during a cognitive task whereas subliminal priming with inaction words led to premature effort withdrawal during the same task (Gendolla and Silvestrini, 2010).

Although it is evident that subliminal priming with visual cues related to affect and action can alter effort during cognitive tasks, the effects of such non-conscious visual cues on perception of effort and endurance performance are currently unknown. Establishing these effects is important for endurance athletes and would build on the recognized associations between affective states and endurance performance (Lane et al., 2010) and the links between action words and basic motor activity such as chewing (Albarracín et al., 2008).

The current investigation consists of two experiments. The aim of Experiment 1 was to establish the effects of subliminal priming with affective facial expressions (happy and sad faces) on rating of perceived exertion (RPE) and endurance performance. The aim of Experiment 2 was to establish the effects of subliminal priming with action and inaction words on RPE and endurance performance. Experiment 1 utilized a traditional group design whereas Experiment 2 employed a single-subject design. The latter was used to illustrate the application of randomization tests to assess the effects of non-conscious psychological manipulations in individual athletes as this approach may have important practical uses (see Section General Discussion). In both experiments a TTE test was used to establish the effects of each subliminal priming procedure on RPE and endurance performance without the confounding effect of individual pacing (Hopkins et al., 2001). This test has previously been shown to be a sensitive measure of endurance performance during cycling exercise (Amann et al., 2007).

## Experiment 1: subliminal priming with happy or sad faces

### Introduction

A clear relationship exists between affective states and sports performance, with positive affect generally associated with better performance and negative affect generally associated with poorer performance (Beedie et al., 2000; Leunes, 2000; Davis et al., 2010). In particular, one aspect of this research has established that affect is related to endurance performance (Lane et al., 2010; Renfree et al., 2011). Despite the widely accepted link between affect and endurance performance (Lane et al., 2012; Stanley et al., 2012), the manner in which endurance performance may be influenced by non-conscious affective cues is still unknown. Based on the psychobiological model of endurance performance, the hypotheses of Experiment 1 were that RPE would be reduced and TTE increased when individuals are subliminally primed with happy faces compared to sad faces during cycling exercise.

### Materials and methods

#### Participant characteristics and ethics

Fourteen healthy and recreationally trained individuals volunteered to take part in the study. One female participant was excluded due to a computer malfunction that revealed one affective facial expression during the final visit. Hence 13 participants were included in the final data analysis [7 males, mean ± *SD*, age 20.1 ± 1.5 years, peak power output (PPO) 328 ± 54 W, maximum oxygen uptake ($\overset{\cdot }{\text{V}}{\text{o}}_{2\max}$) 60.4 ± 6.9 ml·kg^−1^ · min^−1^; 6 females, mean ± *SD*, age 21.0 ± 1.6 years, PPO 233 ± 34 W, $\overset{\cdot }{\text{V}}{\text{o}}_{2\max}$ 49.2 ± 6.5 ml· kg^−1^ · min^−1^]. Participants were engaged in endurance exercise on a minimum of one occasion per week. The study was approved by the ethics committee of the School of Sport, Health and Exercise Sciences (SSHES), Bangor University. Accordingly, prior to taking part all participants completed an informed consent form along with a standard medical questionnaire to disclose their present state of health. Before providing informed consent, participants received an overview of the procedures and requirements of the study and were informed that it was a reliability study testing the accuracy of wireless electroencephalography in detecting the neural responses to unanticipated computer stimuli. Consequently, participants were naive to the true aims of the study until its cessation. They were then debriefed and requested not to discuss it further. A payment of £30 (approximately $45/€35) was given to all participants for their involvement.

#### Experimental design and procedures

The experiment consisted of a single blind, randomized and counterbalanced crossover design in which all participants visited the laboratory on four separate occasions. All exercise tests were conducted at the same location, at a similar time of day, on the same electromagnetically braked cycle ergometer (Excalibur Sport, Lode, Groningen, Netherlands). Saddle and handlebar specifications on the cycle ergometer were adjusted to suit the preference of each participant. These specifications were then maintained for every visit thereafter.

During Visit 1, each participant completed the informed consent questionnaire and a checklist to ensure compliance with pre-task instructions; anthropometric measurements were then recorded. After this, an incremental ramp test was carried out on the cycle ergometer to establish PPO and $\overset{\cdot }{\text{V}}{\text{o}}_{2\max}$. The incremental ramp test began with a 2 min rest after which power output was increased by 25 W every minute until exhaustion. Exhaustion was operationally defined as the point at which either the participant voluntarily terminated the test or cadence had fallen below 60 revolutions per minute (RPM) for five consecutive seconds despite strong verbal encouragement. The cycle ergometer was set in hyperbolic mode, which allows the power output to be set independently of pedal frequency over a range of 30–120 RPM and the participant was instructed to remain in the saddle at all times. $\overset{\cdot }{\text{V}}{\text{o}}_{2\max}$, defined as the highest oxygen uptake measured during the test over a 15 s average, was measured breath by breath via a computerized metabolic gas analysis system (Metalyzer 3B, Cortex Biophysik, Leipzig, Germany) connected to an oro-(mouth) mask (7600 series, Hans Rudolph, Kansas City, MO, USA). The device was calibrated before each test using a known concentration of gases and a 3.0 liter calibration syringe (Series 5530, Hans Rudolph). Heart rate was recorded 15 s from the end of the 2 min rest using wireless chest strap radio telemetry (S610, Polar Electro, Kempele, Finland) and was measured every minute during the test thereafter. During the incremental ramp test, subjects were familiarized with RPE (see Section Rating of Perceived Exertion).

Visit 2 was a familiarization session in which participants completed all questionnaires (see Section Psychological Questionnaires) and the TTE test to be used during Visits 3 and 4. Upon arrival for Visit 3 participants completed mood and motivation questionnaires followed by the TTE test. For this test, participants were positioned on the cycle ergometer (set to hyperbolic mode) and instructed to remain in the saddle at all times. The test began with a 3 min warm up at 30% of the participants PPO. After 3 min the power output was automatically increased to a power output corresponding to 65% PPO. Pedal cadence was freely chosen between 60–100 RPM and was recorded every minute during the test, as was heart rate (see Section Additional Physiological Measures). RPE was recorded at 2 min intervals during the test (see Section Rating of Perceived Exertion). TTE was defined as the time accrued from the onset of 65% PPO until the point at which either the participant voluntarily terminated the test or pedal cadence had fallen below 60 RPM for five consecutive seconds. No verbal encouragement was provided at any point during the TTE test to avoid experimenter bias. Furthermore, to avoid bias from facial mimicry, the experimenter stood behind participants at all times (Tassinary et al., 2007). Exactly 3 min after the cessation of the TTE test, participants provided an earlobe sample of whole fresh blood for lactate analysis (see Section Additional Physiological Measures).

Throughout the TTE test participants were exposed to a scanning visual vigilance task (see Section Scanning Visual Vigilance Task). This computerized cognitive task commenced at the onset of the TTE test and stopped at exhaustion. Corresponding to a randomized and counterbalanced order, subjects were allocated to priming with either happy or sad faces which were subliminally delivered within the scanning visual vigilance task for the duration of the TTE test (see Section Subliminal Priming Procedure). During the TTE test the computer screen was placed at eye level 60 cm away from participants. After the TTE test and the blood sample, participants completed the mood questionnaire for the second time.

All procedures during Visit 4 were identical to Visit 3, other than participants being subliminally primed with the alternative affective facial expression. At the end of Visit 4, participants underwent a standardized funneled debriefing procedure (Bargh and Chartrand, 2000). This was to probe for interpretation of the experimental hypotheses and awareness of the subliminal visual cues (see Section Funneled Debriefing Procedure). If any participants alluded to a face within or before the black and white pattern they were further probed for gender, facial expression and any other details. After being fully debriefed, participants were thanked and then received their payment.

The two experimental visits were separated by a minimum of 5 days and a maximum of 14 days. During this time, individuals were instructed to maintain their normal training program. As requested prior to each visit, participants preserved similar dietary patterns during the preceding 24 h while consuming an amount of water equivalent to least 35 ml·kg^−1^ body weight and attaining at least 7 h of sleep the night before. Participants also avoided any heavy exercise in the 24 h prior to testing and refrained from the consumption of caffeine and nicotine 3 h before each exercise test. Finally, participants voided before all exercise tests and performed them in similar clothing during every visit. Participants remained unaware of their TTE for the familiarization visit and for Visits 3 and 4 until the final debriefing procedure.

#### Scanning visual vigilance task

The scanning visual vigilance task developed by Lieberman et al. (1998) was used in order to deliver subliminal visual cues related to affect during the TTE test. As mental fatigue has been shown to affect RPE and endurance performance (Marcora et al., 2009), this task was selected to minimize the cognitive demands imposed upon participants during the TTE test. Participants were therefore simply requested to focus on the computer screen at all times and informed that a green circle of 3 cm diameter would randomly appear somewhere on the screen. The time that elapsed between each appearance of the circle was no shorter than 45 s, and no longer than 90 s. Participants were instructed to carry on cycling whilst maintaining focus upon the screen, but no response was required when the circle appeared. Each 3 cm green circle appeared at an identical time and screen location for all participants across both visits.

#### Subliminal priming procedure

**Figure 1:**
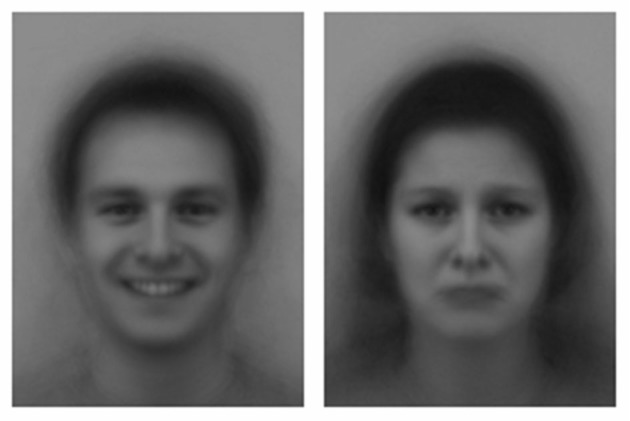
**An example of the happy and sad facial expressions used for subliminal priming during the time to exhaustion test**.

Subjects were subliminally primed during the scanning visual vigilance task throughout each TTE test. One prime was presented sequentially every 4996 ms. Each prime sequence consisted of a white fixation cross that was displayed on a black background in the center of the computer screen (1000 ms). This was instantly followed by a facial expression (16 ms) that was backward masked by a briefly flashed black and white pattern (130 ms). Following the backward mask, the screen either remained black (3850 ms) or alternatively a green circle of 3 cm diameter appeared against the black background in a random location (3850 ms). The next prime sequence was commenced immediately after this. In order to prevent habituation to the affective facial expressions, two thirds of the prime sequences consisted of a neutral face with the remaining one third consisting of the relevant affective facial expression (Silvestrini and Gendolla, 2011a). So as participants were exposed to the affective facial expressions throughout the TTE test, it was ensured that two affective facial expressions were presented within every six prime sequences. The remaining four primes within each six prime sequence consisted of neutral faces. The affective facial expressions consisted of a front perspective black and white human image (see Figure 1) taken from the Averaged Karolinska Directed Emotional Faces (ADKEF) database (Lundqvist and Litton, 1998). Half of the faces were male (MNES, MSAS, MHAS) and the other half were female (FNES, FSAS, FHAS). The priming program was generated in E-prime software (E-Prime, Psychology Software Tools, Pittsburgh, PA, USA) and the primes were presented on a 19′ computer monitor with an aspect ratio of 16:9, a refresh rate of 60 Hz, and a 1280 × 720 pixel array.

#### Funneled debriefing procedure

The funneled debriefing procedure was administered as a manipulation check to ensure that participants were not consciously aware of the affective facial expressions during the subliminal priming procedure. This method was adopted from previous recommendations from subliminal priming research (Bargh and Chartrand, 2000) and consisted of six questions about the subliminal priming procedure. These questions investigated participant perspectives on: (1) the purpose of the experiment; (2) any curiosities during the experiment; (3) the relation between the TTE test and the computer task; (4) the effect of the computer task on the TTE; (5) the reason for the black and white pattern that acted as the backward mask; and (6) anything specific before, during, or after the presentation of the black and white pattern.

#### Rating of perceived exertion

Perception of effort was measured using the Category Ratio 10 (CR10) scale developed by Borg (1998) using standardized instructions and verbal anchors for RPE. Low (0.5) and high (10) anchors were established during the incremental ramp test using standard procedures (Noble and Robertson, 1996). A CR10 scale was provided to all participants after Visit 1, and standardized instructions for RPE were repeated prior to each TTE test with the emphasis that each rating should be based upon the leg effort required to cycle and how heavy is the breathing as opposed to any leg pain or discomfort that may be associated with cycling exercise. Furthermore, participants were informed that they were free to rate perceived exertion above 10 if they perceived effort to be higher than the maximal effort perceived during the incremental ramp test. During the TTE test, the CR10 scale was automatically presented on the computer screen every 2 min. Participants were requested to read out the number that corresponded to their present rating upon every presentation of the scale. For each measurement, the CR10 scale remained on screen for 3850 ms and always replaced the black screen that appeared immediately after the backward mask.

#### Additional physiological measures

Heart rate was recorded throughout the TTE test using wireless chest strap radio telemetry (S610, Polar Electro, Kempele, Finland). Before testing, the chest strap was dampened and securely fastened to the participant’s chest according to the manufacturer’s guidelines. Lactate concentration was measured by collecting 5 μl of whole fresh blood from the earlobe 3 min after the TTE tests. Each blood sample was immediately analyzed using a calibrated device (Lactate Pro LT-1710, Arkray, Shiga, Japan).

#### Psychological questionnaires

In order to evaluate differences in conscious affective state before and after the TTE test, mood was assessed using two self-reported items of the positive (*happy and joyful*) and negative (*sad and depressed*) hedonic tone scales of the U-WIST mood adjective checklist developed and validated by Matthews et al. (1990). The four mood items were rated according to momentary affective state (*right now, I’m feeling*) on a 7-point Likert-type scale ranging from (1) *not at all*, to (7) *very much*. These items have been used elsewhere in order to establish conscious affective state during similar subliminal priming studies (Silvestrini and Gendolla, 2011b; Freydefont et al., 2012). Consistent with this previous research, a global mood rating was established by summating the positive hedonic tone scale items with the reverse scored negative hedonic tone scale items. Motivation was measured prior to the TTE test via the success motivation (example item; *The task will bring out my competitive drive*) and intrinsic motivation (example item; *Doing the task is worthwhile*) scales developed and validated by Mathews et al. (2001). Each scale consists of seven items responded to on a 5-point Likert-type scale (0 = *not at all*, 1 = *a little*, 2 = *moderately*, 3 = *quite a bit*, 4 = *extremely*).

#### Statistical analyses

Unless otherwise noted, data are shown as mean ± *SD*. All data were first checked for normality using the Shapiro-Wilk test. Following this, paired sample *t*-tests were used to assess the effect of visit order on TTE, and the effects of subliminal affective priming on TTE, mean cadence, pre-exercise global mood rating, intrinsic motivation, success motivation, and various measures at exhaustion (RPE, heart rate, and blood lactate concentration). A 2 × 2 (Condition × Time) fully repeated measures analysis of variance (ANOVA) was used to assess the effects of subliminal affective priming on global mood ratings measured before and after the TTE test. A 2 × 5 (Condition × Iso-time) fully repeated measures ANOVA was used to assess the effects of subliminal affective priming on RPE and heart rate measured at 0%, 25%, 50%, 75% and 100% of iso-time during the TTE test. To obtain these iso-time data, the value of each parameter at 100% iso-time was established by identifying the shortest TTE accomplished by each individual over their two tests. The value for each variable attained during the final full minute of the shortest TTE test was then compared to the value attained during the equivalent minute of the longer TTE test. The minute identified as 100% iso-time was multiplied by 0.5 and rounded to the nearest time of rating where necessary to attain the value corresponding to 50% iso-time. To establish 25% iso-time values, the minute identified as 100% iso-time was multiplied by 0.25. To establish 75% iso-time the minute identified as 100% iso-time was multiplied by 0.75. Iso-time values for 0% were attained by comparing values for the first full minute of each TTE test. For all ANOVAs, if assumptions of sphericity were violated, the Greenhouse-Geisser correction was used while Tukey’s HSD *post hoc* tests were calculated where appropriate. Standardized Cohen’s *d* values were calculated using Morris and DeShon’s (2002) equation 8 to provide an estimate of effect size. Thresholds for small, moderate, and large effect sizes were set at 0.2, 0.5, and 0.8, respectively (Cohen, 1988). Precision of the estimate was established by ± 90% confidence intervals were relevant (Hopkins et al., 2009). This indicated the plausible range within which the population effect for a measure may reside (Cumming, 2014). For all data analysis, statistical significance was set at *p* < 0.05 (two-tailed) and conducted using the statistical package for social sciences (SPSS version 16).

### Results

#### Manipulation check

During the funneled debriefing procedure, the description given by all participants regarding their understanding of the study rationale conformed to the study rationale that they were provided with before the experiment. No participant was able to report anything unusual that was related to the real experimental manipulation, no participant suggested that the computer screen affected what they did during the TTE test and no participant was able to decipher the real reason for the black and white patterned backward mask. Three participants mentioned a possible brief facial silhouette on one occasion within the flashed black and white pattern that acted as the backward mask. However when probed further, no participant was able to elaborate on the facial expression of this silhouette. As no participant was suspicious of the study’s true purpose at its culmination and no participant was aware of any facial expression within or prior to the flashed backward mask, this was considered to provide sufficient evidence of a successful experimental manipulation.

#### Effects of subliminal affective priming on mood and motivation

We created separate global mood ratings for pre-exercise and post-exercise mood by adding the positive hedonic tone scale items to the reverse scored negative hedonic tone scale items (Table 1). The global mood ratings between conditions were not significantly different pre-exercise, *t*_(12)_ = 0.48, *p* = 0.64. In addition, neither subliminal affective priming (main effect of condition: *F*_(1,12)_ = 0.73, *p* = 0.41) nor the TTE test (main effect of time: *F*_(1,12)_ = 0.17, *p* = 0.69) affected the global mood ratings. There was also no significant condition x time interaction on this measure of mood (*F*_(1,12)_ = 0.14, *p* = 0.71). These findings indicate that subliminal affective priming did not affect conscious affective state either before or after the TTE test.

**Table 1:** **Mean ± *SD* of global mood ratings for U-WIST mood adjective checklist**.

	Pre-exercise	Post-exercise
Happy	23.2 ± 2.6	23.6 ± 2.7
Sad	22.9 ± 2.7	23.0 ± 2.3

Similar to mood, ratings for success motivation (happy faces = 18.4 ± 5.1, sad faces = 17.1 ± 5.6, *t*_(12)_ = 1.73, *p* = 0.11) and intrinsic motivation (happy faces = 23.2 ± 3.1, sad faces = 22.2 ± 2.6, *t*_(12)_ = 1.95, *p* = 0.07) were not statistically different between conditions.

#### Effect of subliminal affective priming on TTE

**Figure 2:**
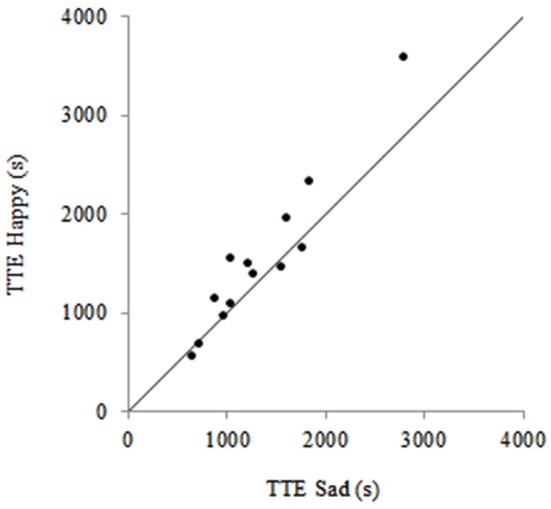
**Scatterplot showing individual time to exhaustion (TTE) data following subliminal priming with happy faces and subliminal priming with sad faces**. The points above the identity line represent a greater TTE following subliminal priming with happy faces compared to sad faces.

As predicted, subliminal affective priming had a significant effect on TTE, with participants cycling for 178 s (12%) longer when they were subliminally primed with happy faces (1519 ± 787 s) in comparison to when they were subliminally primed with sad faces (1342 ± 585 s), *t*_(12)_ = −2.28, *p* = 0.04, *d* = 0.88, 90% CI [38 s, 318 s]. As shown in the condition-by-condition scatterplot (see Figure 2), eight individuals performed greater on the TTE when they were subliminally primed with happy faces compared to sad faces. No order effect was present for TTE across visits, *t*_(12)_ = −0.65, *p* = 0.53.

#### Effects of subliminal affective priming on mean cadence and heart rate, blood lactate concentration and RPE at exhaustion

At exhaustion, there were no significant differences between subliminal priming with happy and sad faces in heart rate, blood lactate concentration, and RPE (Table 2). The latter finding indicates that participants were willing to exert a maximal effort in both conditions. Mean cadence was however greater when participants were subliminally primed with happy faces compared to when subliminally primed with sad faces. Albeit statistically significant, this difference would be considered marginal.

**Table 2:** **Mean ± *SD* of mean cadence, and heart rate, blood lactate concentration and RPE at exhaustion**.

	Sad	Happy	*p*-value
Blood [la] (mmol·l)	7.13 ± 1.86	6.97 ± 2.51	0.60
Heart Rate (beats·min^−1^)	180 ± 10	181 ± 10	0.53
RPE (0–10+)	9.92 ± 0.28	9.96 ± 0.32	0.33
Mean Cadence (RPM)	75.8 ± 4.7	78.0 ± 3.3	0.01

*Note. Blood [la] = blood lactate concentration; RPE = rating of perceived exertion; RPM = revolutions per minute*.

#### Effects of subliminal affective priming on heart rate and RPE during the TTE test

**Figure 3:**
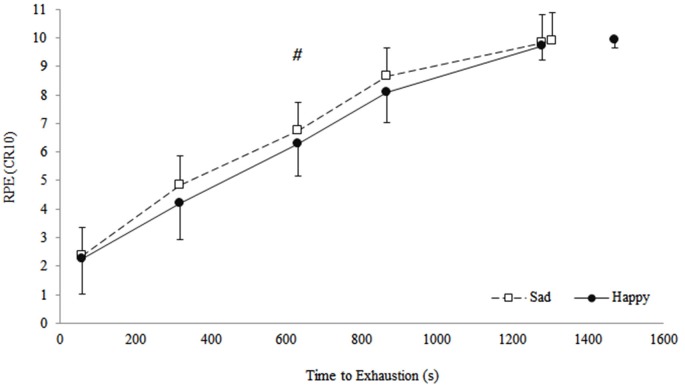
**Effect of subliminal priming with happy or sad faces on rating of perceived exertion (RPE) at 0%, 25%, 50%, 75%, 100% iso-time, and at exhaustion during the time to exhaustion test**. Data are presented as mean (± *SD*). # Indicates significant main effect of condition at iso-time (*p* = 0.04).

RPE increased significantly during the TTE test (main effect of iso-time: *F*_(2.13,25.50)_ = 152, *p* = 0.001). However, RPE was significantly lower following subliminal priming with happy faces compared to subliminal priming with sad faces (main effect of condition: *F*_(1,12)_ = 5.29, *p* = 0.04). Despite this, no significant condition × iso-time interaction (*F*_(4,48)_ = 1.43, *p* = 0.24) was evident (see Figure 3).

Heart rate increased significantly during the TTE test (main effect of iso-time: *F*_(1.22,14.69)_ = 127, *p* < 0.001) however there was no significant main effect of condition (*F*_(1,12)_ = 0.19, *p* = 0.67) or condition x iso-time interaction (*F*_(1.98,23.74)_ = 1.08, *p* = 0.36) with a mean heart rate of 171 ± 10 beats·min^−1^ when participants were subliminally primed with happy faces, compared to a mean of 170 ± 10 beats · min^−1^ when participants were subliminally primed with sad faces.

### Discussion

The findings that non-conscious visual cues related to affect can alter perception of effort and performance during whole-body endurance exercise are in line with previous studies that have effectively used similar subliminal priming procedures to manipulate performance (Freydefont et al., 2012) and effort (Silvestrini and Gendolla, 2011b) during cognitive tasks. The fact that the positive effect of subliminal priming with happy faces on TTE was associated with a reduction in RPE when compared to subliminal priming with sad faces is also consistent with the psychobiological model of endurance performance which posits that, in well-motivated individuals, the primary factor determining endurance performance is perception of effort.

The present findings provide experimental evidence to support the hypothesis that positive affect is associated with better sport performance and negative affect is associated with poorer sport performance. This is important as support to this hypothesis is seemingly limited to correlational findings (Lane et al., 2012; Stanley et al., 2012) and indirect experimental manipulations (Astorino et al., 2012; Terry et al., 2012). As such, the present research provides the first evidence that affective cues can influence perception of effort and endurance performance when they are manipulated directly. Even more importantly, the current findings considerably extend this research area to show that perception of effort and endurance performance can be altered by affective visual cues that occur outside of conscious awareness.

## Experiment 2: subliminal priming with action or inaction words

### Introduction

The aim of Experiment 2 was to replicate the findings of Experiment 1 with a different type of subliminal visual cues: action and inaction words. This type of non-conscious visual cues is relevant for a number of reasons. Specifically, the effects of subliminal priming with action or inaction words on perception of effort and endurance performance may be mediated by alterations in pre-motor and motor areas of the brain. Activity of these cortical areas during muscle contractions is associated with perception of effort in humans (de Morree et al., 2012) and action words have shown to activate premotor and motor areas of the brain (Hauk et al., 2004) showing that the cortical systems for language and action are reciprocally connected (Pulvermüller, 2005). Supportively, in comparison to subliminal inaction words, subliminal action words have been shown to prompt greater activity on basic motor tasks such as chewing (Albarracín et al., 2008). Moreover, subliminal action and inaction words have recently been shown to alter effort during cognitive tasks (Gendolla and Silvestrini, 2010). Because perception of effort is thought to be generated by neurocognitive processing of corollary discharges from premotor and/or motor areas of the brain (Marcora, 2009; de Morree et al., 2012), evidence that action words can affect these cortical areas (Hauk et al., 2004) provides some neurobiological rationale for an effect of subliminal action words on perception of effort and, thus, endurance performance. To test these hypotheses, Experiment 2 implemented a relatively novel single subject experimental design (Dugard et al., 2012) to assess the effects of subliminal priming with action and inaction words on perception of effort and endurance performance during cycling exercise. Therefore, the hypotheses of this experiment were that RPE would be reduced and TTE increased when individuals are subliminally primed with action words compared to inaction words.

### Materials and methods

#### Participant characteristics and ethics

One healthy male participant (age 22 years, PPO 287 W, $\overset{\cdot }{\text{V}}{\text{o}}_{2\max}$ 58.3 ml·kg^−1^ · min^−1^) volunteered to take part in the study. The participant was an experienced endurance athlete having trained and competed at a regional level in endurance sports for 4 years at the time of the study. Accordingly the participant was regularly engaged in endurance exercise (running and cycling) on at least two occasions per week and continued to also frequently take part in competitive endurance events. The participant received £150 (approximately $250/€180) for his involvement in the study. The study was approved by the ethics committee of the SSHES, Bangor University. Prior to taking part the participant completed an informed consent form along with a standard medical questionnaire to confirm his present state of health. The participant was provided with a detailed overview of all procedures and requirements of the study before its commencement and was informed that the study was a reliability study designed to test the ability of wireless electroencephalography to accurately detect the neural responses to unanticipated computer stimuli. Consequently, the participant was naive to the true aims and hypotheses until the cessation of the study, at which point he was debriefed about its genuine rationale.

#### Experimental design

The experiment was a single blind, blocked randomization tests design (Dugard et al., 2012) in which the participant visited the laboratory on 14 occasions. Visits 1 and 2 involved an incremental ramp test and a familiarization session respectively and Visits 3 to 14 were comprised of the 12 experimental visits. These 12 experimental visits encompassed a crossover design in which the participant was randomly allocated to 6 visits for each of the two experimental treatment conditions (subliminally primed action vs. inaction words). The order of treatment visits was randomized in three blocks of four, categorized in sequence as Blocks 1, 2, and 3. Accordingly, within each block the individual was randomly allocated to two visits for each treatment. This blocked procedure was performed to provide further control over the training effect that consecutive TTE tests may elicit along with other confounding order effects such as learning and demotivation (Dugard et al., 2012). The blocks of randomized visits were merged in numerical sequence to create one complete experimental arrangement of treatments across the 12 visits (*Block 1: action, inaction, inaction, action; Block 2: inaction, inaction, action, action; Block 3: inaction, action, inaction, action*).

#### Procedures

All exercise tests were conducted at the same location, at a similar time of day, on the same electromagnetically braked cycle ergometer (Excalibur Sport, Lode, Groningen, Netherlands). Saddle and handlebar specifications on the cycle ergometer were adjusted on the first visit to suit participant preference and these specifications were then maintained for every visit. Visit 1 consisted of an incremental ramp test to establish PPO and $\overset{\cdot }{\text{V}}{\text{o}}_{2\max}$. All procedures for this incremental test were identical to those in Experiment 1. Visit 2 consisted of a familiarization session in which the participant completed all questionnaires (see Section Psychological Questionnaires) and the TTE test that was to be used during the 12 experimental visits. This familiarization session was also identical to the familiarization session outlined in Experiment 1. For the subsequent 12 visits, upon arrival the participant first completed mood and motivation questionnaires (see Section Psychological Questionnaires), this was followed by the TTE test. Other than the randomly allocated subliminal word primes delivered during the 12 TTE tests, the procedure for all 12 of these tests was identical to the TTE tests used in Visits 3 and 4 of Experiment 1. At the end of Visit 14 the participant underwent a standardized funneled debriefing procedure (Bargh and Chartrand, 2000) to probe for interpretation of the experimental hypotheses and awareness of the subliminal word primes. After being fully debriefed the participant was thanked and then received his payment.

Visits 1, 2, and 3 were separated by a minimum of 7 days each, while all visits between Visits 3 and 14 were separated by a minimum of 6 days and a maximum of 15 days. All pre-visit instructions were the same as those for Experiment 1. The participant remained unaware of his TTE value for the familiarization visit and for every subsequent visit until the final debriefing procedure.

#### Subliminal priming procedure

In conjunction with visit allocation, action or inaction words were subliminally primed within the computerized scanning visual vigilance task for the duration of each TTE test. The procedure for the scanning visual vigilance task was identical to that of Experiment 1 other than the fact that subliminally primed words replaced the subliminally primed affective facial expressions that were used in Experiment 1. During the subliminal priming procedure, a word prime sequence was presented serially every 4996 ms. Each prime sequence first consisted of a white fixation cross that was displayed on a black background in the center of the screen (1000 ms). This was instantly followed by a word prime (16 ms) that was backward masked by a random letter sequence (130 ms). This random letter sequence always consisted of the letters MZKGWB and appeared after every word prime.

Following the backward mask, the screen either remained black (3850 ms) or alternatively a green circle of 3 cm diameter appeared against the black background in a random location on the screen (3850 ms). The next word prime sequence commenced immediately after. To prevent habituation to the subliminal word primes, two thirds of the primes consisted of non-word primes with the remaining one third consisting of the word primes (Silvestrini and Gendolla, 2011a). To ensure that exposure to the subliminal word primes occurred throughout each TTE test, two word primes were randomly presented within each six prime sequence. The remaining four primes within each six prime sequence therefore consisted of the non-word primes.

The word primes were obtained from the empirically derived Computerized Edinburgh Associative Thesaurus (Kiss et al., 1973). The selected action primes consisted of the words *ACTION*, *GO*, *LIVELY* and *ENERGY*. The inaction primes consisted of the words *STOP*, *TOIL*, *SLEEP*, and *TIRED*. The non-word primes were created by re-arranging the letter order of the action and inaction words. The word primes, non-word primes, and the backward mask were all presented in white capital letters of size 125 calibri font in the center of the screen and against a black background. The priming program was generated in E-prime software (E-Prime, Psychology Software Tools, Pittsburgh, PA) and the primes were presented on a 19′ computer monitor with an aspect ratio of 16:9, a refresh rate of 60 Hz and a 1280 × 720 pixel array.

#### Funneled debriefing procedure

The funneled debriefing procedure (Bargh and Chartrand, 2000) was the same as Experiment 1 other than specific alterations to Questions 5 and 6 which were adjusted to: (5) the reason for the random letter string that acted as the backward mask; and (6) anything specific regarding the letters.

#### Rating of perceived exertion

The procedures used to measure RPE were identical to those in Experiment 1 except that RPE was measured at 1 min intervals during the TTE test.

#### Psychological questionnaires

The Brunel mood scale (BRUMS) was used to assess mood before the TTE test. This measure of mood has been validated for use with adult populations (Terry et al., 2003). The measure is comprised of six subscales (anger, confusion, depression, fatigue, tension, and vigor) with four items per subscale. Items were answered on a 5-point Likert-type scale (0 = *not at all*, 1 = *a little*, 2 = *moderately*, 3 = *quite a bit*, 4 = *extremely*). The procedures that were used to measure motivation prior to the TTE test were identical to those in Experiment 1.

#### Statistical analyses

Unless otherwise noted, data are shown as mean ± *SD*. Randomization tests (Dugard et al., 2012) were used to assess for mean differences between treatments (action vs. inaction words) in TTE, mean cadence, all BRUMS subscales, success and intrinsic motivation, and various measures at exhaustion (RPE, heart rate, and blood lactate concentration). Randomization tests were also used to assess differences between conditions for RPE and heart rate at the 15th minute of the TTE test. The 15th minute was selected as this represented the final full minute of the shortest TTE over the 12 Visits.

For each randomization test, in order to test for statistical significance, mean values for each treatment condition were first calculated. The difference between these means was then obtained. These values provided the true experimental difference between treatments for each dependent variable. The randomized order of experimental treatments across the 12 visits represented one of many possible ways in which the treatment visits could have been arranged. Using a pre-designed macro (Dugard et al., 2012) the raw data from the 12 experimental treatment visits was randomly rearranged 2000 times to coincide with alternative visits in the original treatment allocation. For each of these 2000 rearrangements, only the raw data from treatment conditions was randomly rearranged with the allocated treatment order of the respective 12 experimental visits remaining the same. Specifically, this meant that the raw data for each visit was randomly swopped between the allocated treatment visits two thousand times. Due to the present design, this procedure was performed only on a within block basis. Hence for example, a raw value from Block 1 could not be rearranged to a visit in Blocks 2 or 3. Rearranging the raw data in proximity to the assigned visits in this manner permitted the calculation of a mean difference between treatment conditions for each of the 2000 treatment rearrangements. In order of magnitude from high to low, the true mean difference was then ranked amongst the 2000 mean differences that were obtained from the treatment rearrangements. Statistical significance was obtained if the mean difference for the experimental data was greater than 95% of the mean differences acquired from the 2000 treatment rearrangements. Statistical significance was set at *p* < 0.05 (one-tailed) for TTE and RPE and *p* < 0.05 (two-tailed) for all other analyses. All data analysis was conducted using a specified macro (Dugard et al., 2012) in Microsoft Excel 2010.

### Results

#### Manipulation check

A qualitative evaluation of the funneled debriefing procedure indicated that the participant believed the cover rationale for the study to be genuine throughout. The participant also did not detect any subliminally primed action and inaction words during the 12 visits. The participant therefore remained naive to the true experimental hypotheses during the investigation.

#### Effects of subliminal priming with action or inaction words on mood and motivation

A randomization test for each BRUMS subscale revealed no significant differences in pre-exercise mood between conditions (see Table 3). Similarly, no significant differences were evident between conditions for ratings of success or intrinsic motivation related to the upcoming TTE test.

**Table 3:** **Mean ± *SD* participant rating for all Brunel Mood Scale (BRUMS) subscales, and success and intrinsic motivation prior to the time to exhaustion test**.

	BRUMS subscales						Motivation	
	Anger	Confusion	Depression	Fatigue	Tension	Vigour	Success	Intrinsic
Action	0.00 ± 0.00	0.00 ± 0.00	0.00 ± 0.00	0.00 ± 0.00	2.83 ± 0.75	14.33 ± 1.97	28.00 ± 0.00	28.00 ± 0.00
Inaction	0.00 ± 0.00	0.00 ± 0.00	0.00 ± 0.00	0.00 ± 0.00	4.17 ± 0.75	14.83 ± 0.98	27.67 ± 0.52	28.00 ± 0.00
*p* =	0.99	0.99	0.99	0.99	0.06	0.49	0.33	0.99

#### Effects of subliminal priming with action or inaction words on TTE, mean cadence, and heart rate, blood lactate concentration and RPE at exhaustion

The participant cycled for 399 s longer when subliminally primed with action words in comparison to inaction words (see Table 4). This difference of 399 s between conditions was ranked within the top 3.6% of means that were obtained from the 2000 alternative random treatment arrangements. TTE was therefore significantly greater following subliminal priming with action words vs. inaction words (*p* = 0.04). To make sure that these findings were not affected by the extreme value observed during Visit 2, a second analysis was conducted by replacing this extreme value with the highest value from the corresponding condition in the same block. Even when using this conservative approach, TTE remained significantly greater following subliminal priming with action words vs. inaction words (*p* = 0.05). Unlike TTE, heart rate, blood lactate and RPE at exhaustion were not significantly different between conditions. Mean cadence during the TTE test was also not significantly different between conditions.

**Table 4:** **Individual data by block, visit order, and subliminal word primes for TTE, mean cadence, and heart rate, blood lactate concentration and RPE at exhaustion**.

Block	Visit	Condition	TTE (s)	HR (beats . min^−1^)	Blood [la] (mmol·l)	Mean Cadence (RPM)	RPE (0-10+)
1	1	A	1772	140	2.2	86.5	10
1	2	I	915	136	2.6	80.6	10
1	3	I	1835	137	3.1	85.1	10
1	4	A	1910	144	2.7	86.9	10
2	5	I	2304	146	2.0	82.3	10
2	6	I	2781	153	2.6	84.8	10
2	7	A	2822	148	1.9	80.7	10
2	8	A	2975	142	2.3	81.9	10
3	9	I	2705	139	1.9	81.1	10
3	10	A	3291	138	2.4	81.0	10
3	11	I	2528	139	2.4	82.2	10
3	12	A	2692	136	1.9	81.9	10
**Action (Mean ± SD)**			2577 ± 605	141 ± 4	2.23 ± 0.31	83.1 ± 1.4	10 ± 0
**Inaction (Mean ± SD)**			2178 ± 706	142 ± 7	2.43 ± 0.44	82.7 ± 1.7	10 ± 0

*Note. TTE = time to exhaustion; HR = heart rate; Blood [la] = blood lactate concentration; RPM = revolutions per minute; RPE = rating of perceived exertion; A = action words; I = inaction words*.

#### Effects of subliminal priming with action or inaction words on RPE and heart rate during the TTE test

**Figure 4:**
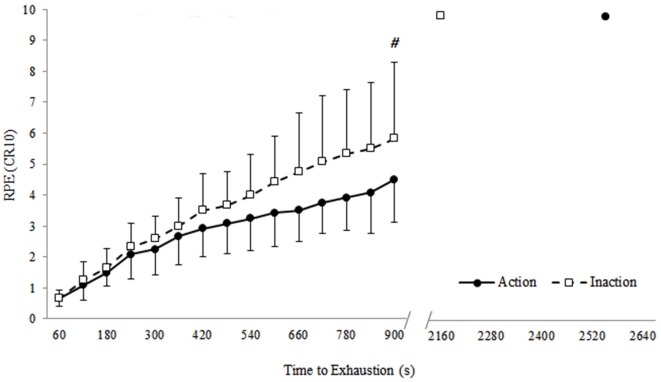
**Effect of subliminal priming with action or inaction words on rating of perceived exertion (RPE) at minute 15 of the time to exhaustion tests and at exhaustion**. Data are presented as mean (± *SD*). # Indicates significant difference between conditions (*p* = 0.03).

Randomization tests between conditions at iso-time (15th minute) revealed that RPE was significantly lower (*p* = 0.03) when the participant was subliminally primed with action words during the TTE test compared to inaction words (see Figure 4). In contrast to RPE, randomization tests revealed that heart rate was not significantly different between conditions at iso-time (*p* = 0.35). Furthermore, there was no significant difference in mean heart rate during the TTE test between conditions (*p* = 0.30) with the participant obtaining a mean heart rate of 138 ± 4 beats·min^−1^ when subliminally primed with action words, compared to a mean of 139 ± 8 beats·min^−1^ when subliminally primed with inaction words.

### Discussion

The results of Experiment 2 bolster those of Experiment 1 by demonstrating that non-conscious visual cues related to action were able to significantly alter RPE and TTE for the individual involved in this single-subject randomized experiment. These findings are consistent with the previously reported effects of subliminal priming with action or inaction words on effort during cognitive tasks (Gendolla and Silvestrini, 2010) and provide the first evidence that these effects extend to physical tasks. In addition, this study exhibits the utility of randomization tests as an effective methodological approach to analyse data from single-subject experiments. Such an approach has worthwhile practical and research based implications for scenarios that involve special populations such as elite athletes where assessment of individual responses is essential.

## General discussion

The purpose of the present research was to investigate the effects of non-conscious visual cues on perception of effort and endurance performance. The two different types of visual cues utilized in Experiments 1 and 2 respectively were able to alter both perception of effort and endurance performance during cycling exercise. Specifically, as hypothesized in Experiment 1, subliminally priming participants with happy faces as they cycled to exhaustion at 65% PPO significantly reduced RPE in comparison to subliminal priming with sad faces. Correspondingly TTE was significantly greater when participants were subliminally primed with happy faces. Similarly, the findings from the single subject approach used in Experiment 2 demonstrated that subliminal priming with action words significantly reduced RPE and enhanced TTE in comparison to subliminal priming with inaction words.

The findings that non-conscious visual cues can affect perception of effort and whole-body endurance performance extend previous reports that non-conscious psychological manipulations have significant effects on effort and behavior during both cognitive and physical tasks (Bargh et al., 2001; Hodgins et al., 2006; Pessiglione et al., 2007; Aarts et al., 2008; Bijleveld et al., 2010; Banting et al., 2011; Silvestrini and Gendolla, 2011b; Freydefont et al., 2012). Gendolla (2000) has previously suggested that affective states influence the appraisal of task demand and the subjective experience of task-related effort. For example, positive moods have been found to reduce appraisals of task demand and experienced effort whereas negative moods have the opposite effects (Gendolla et al., 2001). This corresponds to the predictions made by motivational intensity theory (Brehm and Self, 1989; Wright, 2008). Consequently, lower appraisals of task demand coincide with a reduced experience of effort during objectively easy tasks, whereas effort is more willingly tolerated as task difficulty increases. Conversely, increased appraisals of task demand coincide with an elevated experience of effort during objectively easy tasks, whereas effort is more readily withheld as task difficulty increases (Gendolla et al., 2001). The effect of action and inaction words has also been placed within the context of motivational intensity theory such that action and inaction words are proposed to influence effort as long as task success is regarded as possible and worthwhile (Silvestrini and Gendolla, 2013). Hence primed action words elicit greater effort investment than primed inaction words on cognitive tasks providing that the demands of the task are regarded as possible.

Based on motivational intensity theory, the psychobiological model of endurance performance (Marcora, 2008; Marcora and Staiano, 2010) proposes that individuals will persist with endurance exercise until they reach the maximum amount of effort that they are willing to exert in order to succeed in the task (i.e., potential motivation), or until continuation in the task is perceived as impossible. When one of these two scenarios occurs, the conscious sensation of effort will therefore prompt individuals to voluntarily terminate endurance exercise. In conjunction with the findings of Gendolla et al. (2001) and Silvestrini and Gendolla (2011b), the subliminal priming of happy faces in Experiment 1 therefore likely reduced subjective appraisal of task demand whereas the subliminal priming of sad faces elicited the opposite effect. Consequently, this delayed the point at which a very high perception of effort made continuation in the TTE test seem impossible. In Experiment 2, due to the fixed power output used in the TTE test, it is impossible for individuals to intentionally increase effort. In our study it is therefore likely that action word priming instead permitted the individual to cycle at the same power output for a lower RPE. Alternatively, or concurrently, it is possible that inaction word priming increased the momentary RPE. In either case this would alter the point at which continuation in the task appeared impossible, hence explaining the difference in TTE.

This psychobiological model of endurance performance provides a single theoretical framework to explain the effects of many different physiological and psychological factors known to affect endurance performance. These factors include muscle fatigue (Marcora et al., 2008), muscle damage (Marcora and Bosio, 2007), mental fatigue (Marcora et al., 2009), motivational self-talk (Blanchfield et al., 2014), psychostimulants (Sgherza et al., 2002; Jacobs and Bell, 2004), sleep deprivation (Martin, 1981), inspiratory muscle fatigue (Gething et al., 2004), nutritional supplementation (Blackhouse et al., 2005), and aerobic training (Ekblom and Goldbarg, 1971). Indeed, in all the above examples, changes in endurance performance were associated with changes in RPE. The present findings extend the explanatory power of this model by showing that non-conscious psychological factors may also modify perception of effort and influence the associated decision making process that determine endurance performance.

The significant effects of subliminal visual cues on TTE also challenge the proposal that endurance exercise terminates when the fatigued neuromuscular system (Amann and Dempsey, 2008), or the muscles themselves (Allen et al., 2008; MacIntosh and Shahi, 2011), are no longer able to produce the power/force required by exercise as postulated by the muscle fatigue model of endurance performance. Specifically, it is unlikely that differences in TTE between subliminal priming conditions were mediated by the cardiovascular and metabolic factors commonly associated with muscle fatigue because no significant differences were evident in heart rate or post-exercise lactate in each experiment. Moreover, although mean cadence was different between conditions in Experiment 1, such a marginal difference of two RPM is unlikely to have contributed to changes in motor unit recruitment or cycling efficiency (Dantas et al., 2009). In addition to our physiological measures, pre-exercise mood and motivation were similar between conditions. These findings suggest that these psychological factors did not mediate the effects of subliminal priming on endurance performance. Furthermore, although muscle fatigue was not measured in the present studies, the very nature of subliminal visual cues and their lack of effects on the physiological responses to endurance exercise suggest that an effect on peripheral fatigue is very unlikely. Similarly, there is evidence that cognitive tasks much more demanding than the cognitive task used to provide subliminal visual cues to our subjects do not induce central fatigue (Pageaux et al., 2013). Overall, we believe it is safe to speculate that central and/or peripheral muscle fatigue are unlikely to explain the significant effects of subliminal visual cues on endurance performance, and that a more plausible mechanism is alterations in perception of effort.

The present findings also provide further evidence against one of the main hypotheses of the central governor model of endurance performance: the subconscious brain, based on interoception and previous experience, calculates the maximum time a person can exercise without a catastrophic failure of homeostasis, and regulates RPE and TTE accordingly (St Clair-Gibson and Noakes, 2004). In this regard it is important not to confuse the well-established fact that the brain is capable of processing subliminal visual cues (Pessiglione et al., 2007; Aarts et al., 2008; Silvestrini and Gendolla, 2011b) with the proposal that a subconscious intelligent system regulates neural recruitment of locomotor muscles during endurance exercise to avoid harm to the human (Noakes, 2000; St Clair-Gibson and Noakes, 2004). As shown in the present experiments, subliminal visual cues had significant effects on the conscious sensation of effort which, according to the psychobiological model of endurance performance, determined the different times at which our subjects consciously decided to terminate endurance exercise. On the other hand, it seems highly unlikely that providing subliminal visual cues could affect the physiological condition of the body before and during endurance exercise and, as a result, influence the subconscious and teleoanticipatory calculations made by the central governor about the maximum time our subjects could exercise without a catastrophic failure of homeostasis (Noakes, 2011). Once more, this speculation is supported by the fact that subliminal visual cues did not affect the physiological responses to endurance exercise in our two studies. Therefore, the central governor model does not provide a plausible explanation for the significant effects of subliminal visual cues on perception of effort and endurance performance.

Given the pivotal role played by perception of effort in mediating the effects of subliminal visual cues on endurance performance, it is important to establish the mechanisms that may be responsible for changes in perception of effort during the exposure to subliminal visual cues. Although the present experiments were intended to be exploratory and not designed for this purpose, some potential psychological explanations for the current findings are worthy of consideration. For instance, within the framework of the broaden and build hypothesis (Fredrickson, 2001), positive emotions have been found to broaden the scope of attention and thought-action repertoires compared to a neutral condition, whereas negative emotions narrow thought action repertoires (Fredrickson and Branigan, 2005). Broadening the scope of attention may therefore facilitate attentional dissociation, which has been found to reduce RPE (Lind et al., 2009). Similarly, broadening the scope of thought-action repertoires via the implementation of positive affect may theoretically aid in the activation of task relevant mental representations. In particular it has been proposed that the activation of these mental representations prompts the behavioral effects that occur following the exposure to non-conscious visual cues (Dijksterhuis et al., 2005). With regards to affective cues this is associated with the non-conscious activation of emotion concepts (Niedenthal et al., 2009). These emotion concepts represent an individual schema of memories, motivations and behaviors that surround a precise emotion (Lang et al., 1998). Pertinently, when emotion concepts are activated by a specific affective cue, they are proposed to elicit a behavioral response that is associated with the specific cue (Zemack-Rugar et al., 2007; Silvestrini and Gendolla, 2011b). As such, subliminal priming with visual cues related to happiness and sadness may have respectively activated the concepts of ease and difficulty that have been associated with these emotions (Silvestrini and Gendolla, 2011b; Gendolla, 2012). Similarly, Gendolla and Silvestrini (2010) have proposed that the priming of general action and inaction words can activate matching effort related mental representations. For example, these words are proposed to represent goal motivated end states such that the priming of action words activates the goal to pursue contextually relevant active behavior whereas inaction words activate the goal of inaction and thus task termination (Albarracin et al., 2011). The activation of mental representations of ease or action may therefore have facilitated the lower RPE that allowed individuals to cycle for longer before they voluntarily terminated exercise. Alternatively, activating the mental representations of difficulty or inaction may have contributed to premature task termination by surreptitiously elevating RPE.

In conjunction with the theme of non-consciously activated mental representations, it has also been reported that the sensitivity to non-conscious reward priming increases as effort becomes more pronounced (Bijleveld et al., 2012). As such it is possible that the non-conscious visual primes became more influential as the TTE test progressed and RPE correspondingly increased. Intuitively, such an effect would progressively intensify the desire for task termination when paired with sad faces or inaction words while a prolonged willingness to continue with the task would be expected at high levels of effort when paired with happy faces or action words.

In addition to the similar pre-exercise mood ratings, conscious appraisals of mood did not change from pre to post-exercise and were not different between affective priming conditions. These similarities in conscious mood ratings might initially imply that the subliminal manipulation of affect in the present study was not effective. However, the present finding is consistent with other studies that have utilized non-conscious affective priming (Winkielman and Nowak, 2005; Zemack-Rugar et al., 2007; Silvestrini and Gendolla, 2011b). In these studies it has been suggested that not only are non-conscious affective cues able to modify affective states, but that the affective state itself is also not consciously experienced. This phenomenon has again been attributed to the non-conscious activation of mental representations such that behavioral alterations that are aligned with changes in affect occur despite the inability of participants to consciously report a change in affective state (Zemack-Rugar et al., 2007). As conscious affect was not measured during the TTE test while the subliminal priming procedure took place however, this is currently a hypothetical consideration. Nonetheless, the concept that endurance performance can be altered even when a change in affective state is not consciously perceived does impart an intriguing direction for future research. Further research is also required to understand the neural mechanisms underlying the effects of non-conscious visual cues related to affect and action on perception of effort and endurance performance. Particular clarification might be gleaned from placing emphasis on the anterior cingulate cortex. This cortical area is associated with perception of effort in humans (Williamson et al., 2002) and effort-based decision-making in both non-human animals and humans (Kurnaiwan et al., 2011) and shows greater activation when humans are subliminally primed with happy faces compared to sad faces (Killgore and Yurgelun-Todd, 2004); hence providing a plausible mechanism for the link between subliminal cues and effort.

From a practical perspective, the importance of the present findings are best signified when their effects are compared to the effects of physiological factors known to alter endurance performance. For instance the 12% difference in TTE resulting from subliminal priming with happy or sad faces and the 17% difference in TTE between subliminal priming with action and inaction words can be likened to the negative effects of inspiratory muscle fatigue (14%; Wüthrich et al., 2013), and locomotor muscle fatigue (18%; Marcora et al., 2008). Placing the present findings in this context emphasizes the implications for endurance athletes who may be exposed to non-conscious visual cues during training or competition. Furthermore, because perception of effort is considered to represent one of the main barriers to exercise (Bauman et al., 2012), non-conscious visual cues related to affect and action may also effect exercise adherence in a similar manner.

Despite the theoretical and practical implications of the present experiments, it is also important to recognize some potential limitations. For instance, because a control condition was not utilized in either experiment it is not possible to establish whether the difference in TTE resulted from an increase in endurance performance following subliminal priming with happy faces or action words, a decrease in endurance performance following subliminal priming with sad faces or inaction words, or a combination of both effects. However, it was decided that establishing the existence of an overall effect of non-conscious visual cues on endurance performance was the most pertinent aim of each of these proof of principle studies. Hence, on this occasion, the use of one experimental condition and a control condition was not implemented. In addition, due to the crossover design of both investigations, a forced prime recognition check was not carried out at the culmination of each study. This was again however a known consequence of each methodological design. Specifically, while it is acknowledged that this manipulation check is important, in this instance it was reasoned that a within participant design would more effectively answer our research questions and that this approach would confound such a check. This is because implementing a forced prime recognition check only after the final subliminal priming visit may have introduced recall bias owing to the fact that participants would have been required to recall facial expressions not only for the task that they had just completed, but also for those of the previous visit(s).

In the second experiment a single subject design was implemented to exhibit the use of randomization tests as an important and effective methodological approach to single subject research. It should be noted however that randomization tests lack any ecological validity beyond that of the individual investigated. Wider generalization of the present findings is therefore not possible. Nonetheless, the strength of these tests also resides in this focused individual approach. Importantly, the repeated assessment approach that is inherent in Experiment 2 also permits some interpretation of the manner in which the priming intervention itself may have worked. For instance, the findings hint at the possibility that the effects of action and inaction word priming is acute as opposed to long lasting. As such this approach may be repeatedly used to acutely manipulate performance. It should also be noted that the TTE in Visit 2 was markedly shorter than all others. This occurred despite the participant having adhered to all their instructions prior to the visit. Although the data from this visit may be a genuine result of the treatment manipulation, and should not be regarded as otherwise, it is also acknowledged that it could be anomalous. To account for this the raw performance value for this visit was substituted with the highest TTE value of the corresponding visit within that block. Importantly, despite doing so the difference between conditions remained significant.

Notwithstanding the methodological considerations, obvious directions for future research include the addition of a control condition to the present design to help establish the directional effects of the non-conscious visual cues used in Experiments 1 and 2. Likewise, replicating Experiment 2 from a group based perspective would facilitate wider generalization regarding the effects on non-conscious action and inaction words on RPE and endurance performance. Such approaches might clarify whether non-conscious visual cues can be used as a performance-enhancing strategy during training and competitions in endurance athletes, e.g., by using contemporary technology such as smart glasses. This would also establish whether non-conscious visual cues may be an effective tool to reduce perception of effort and, thus, improve exercise adherence in recreational exercisers. In addition, it would be useful to assess whether these subliminal priming effects remain when individuals are aware that they are being subliminally primed as this would mitigate the ethical considerations associated with subliminal priming. Moreover, the investigation of pre-event subliminal priming may permit a direct approach to performance enhancement in competitive events by eliminating the necessity for visual display technology during the event itself. Finally, the impact of alternative visual cues such as anger or disgust may provide further invaluable insights into some of the factors that non-consciously influence endurance performance.

In conclusion, the collective findings of Experiments 1 and 2 are the first to show that subliminal visual cues can influence perception of effort and endurance performance. These novel findings corroborate the suggestion that endurance performance is regulated by psychobiological factors. Together, this supports and extends the explanatory power of the psychobiological model of endurance performance and the suggestion that any physiological or psychological factor affecting perception of effort and/or potential motivation will affect endurance performance (Marcora et al., 2008). Practically, the present research also has considerable implications for individuals who may be subjected to non-conscious visual cues during recreational exercise, physical training, and competitive endurance events. The fact that this finding occurred in two different experimental contexts therefore highlights the potential strength of non-conscious psychological manipulations and provides a robust platform to further investigate the role played by non-conscious visual cues during endurance performance. As such, future emphasis should be placed on non-conscious strategies designed to ensure optimal performance in endurance athletes and improve exercise adherence in the general population at both a group level and on an individually tailored basis.

## Authors contribution

Anthony Blanchfield, James Hardy, and Samuele Marcora contributed to the conception and design of the work. Acquisition was performed by Anthony Blanchfield and analysis and interpretation of the work was conducted by Anthony Blanchfield, James Hardy, and Samuele Marcora. Anthony Blanchfield, James Hardy, and Samuele Marcora were involved in all stages of the drafting and revision of the work as well as final approval of the version to be published. All authors (Anthony Blanchfield, James Hardy, and Samuele Marcora) agree to be accountable for all aspects of the work.

## Conflict of interest statement

The authors declare that the research was conducted in the absence of any commercial or financial relationships that could be construed as a potential conflict of interest.
